# Basal antero-septal anomalous papillary muscle in hypertrophic obstructive cardiomyopathy

**DOI:** 10.1186/1532-429X-17-S1-P300

**Published:** 2015-02-03

**Authors:** George O Angheloiu, Jason W Vokes, Valerie A Pearce, Kathy L DeLong, Bryan E Jacobson, Sharon R Hallstrom, Bobby Byerly, Sally McClain, Robert W Biederman

**Affiliations:** 1 Cardiology, Penn Highlands, Dubois, PA, Dubois, PA, USA; 2 Allegheny General Hospital, Pittsburgh, PA, USA

## Background

We report the presence of an anomalous papillary muscle inserting into the basal antero-septum in patients with hypertrophic obstructive cardiomyopathy (HOCM) investigated by means of cardiac MRI.

## Methods

9 consecutive patients with HOCM and 13 age- and gender-matched controls without this condition were interrogated using a Siemens Espree MRI machine. A diagnosis of HOCM was established on the basis of asymmetric antero-septal left ventricle hypertrophy and systolic anterior motion of the mitral apparatus.

## Results

**Figure 1:**
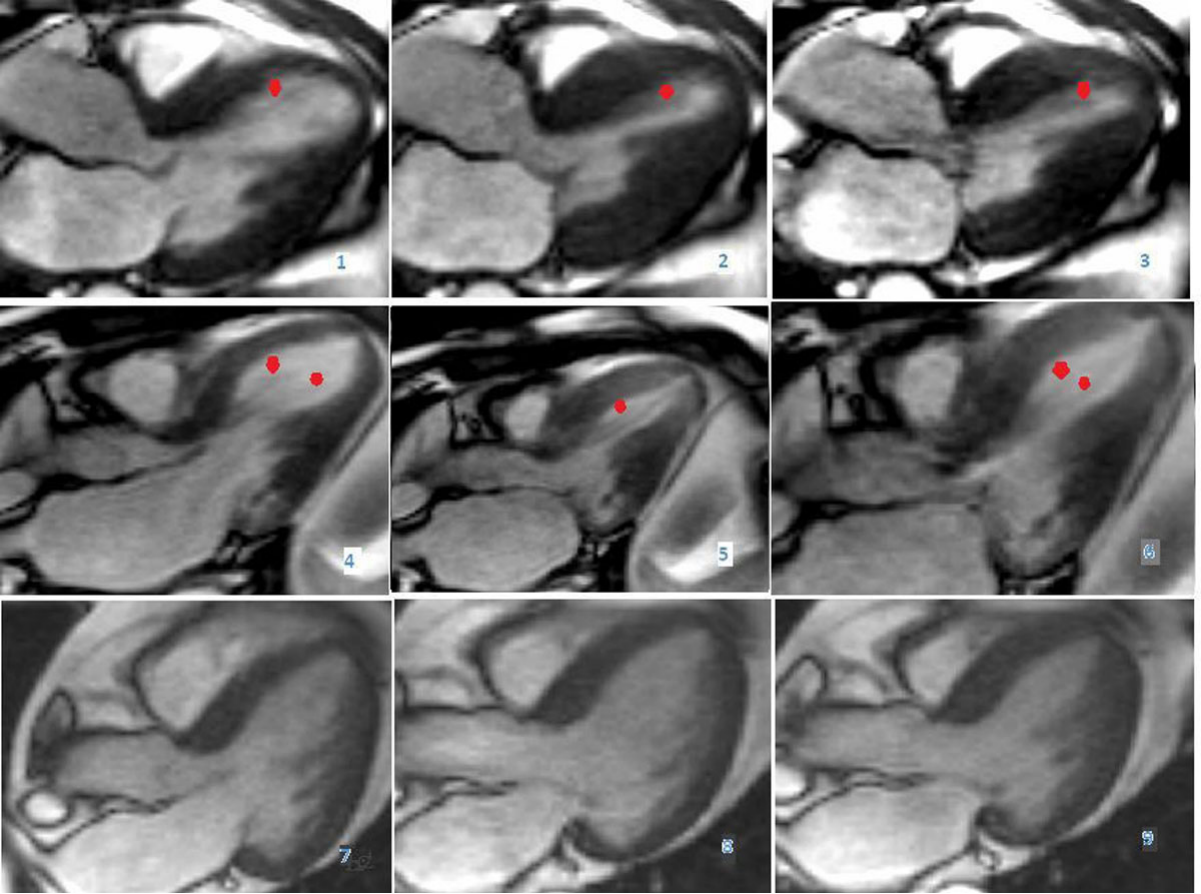


100% of HOCM patients and 62% (n=8, P=0.05) of controls demonstrated an anomalous papillary muscle connecting the left ventricle basal antero-septum with the apex, noticeable on a long axis view acquired on an SSFP sequence. This particular structure contracts during during systole as seen in Figure 1 in panels 1 and 4 (diastole), 2 and 5 (mid-systole) and 3 and 6 (end-systole) in two HOCM patients (panels 1-3 and 4-6 respectively). The papillary muscle was not present in panels 7-9 of one control subject. A prominent and a moderate systolic anterior motion of the mitral apparatus are seen in panels 1-3 and 4-6 respectively.

## Conclusions

An anomalous papillary muscle connecting the basal antero-septum with the left ventricular apex and contracting during systole is more commonly seen in HOCM patients than in controls. Further larger studies are needed to elucidate the importance of this anatomical finding in the physiology of left ventricle outflow tract obstruction.

